# COX-2-Derived Prostanoids and Oxidative Stress Additionally Reduce Endothelium-Mediated Relaxation in Old Type 2 Diabetic Rats

**DOI:** 10.1371/journal.pone.0068217

**Published:** 2013-07-09

**Authors:** Emilie Vessières, Anne-Laure Guihot, Bertrand Toutain, Maud Maquigneau, Céline Fassot, Laurent Loufrani, Daniel Henrion

**Affiliations:** 1 Department of Integrated Neurovascular and Mitochondrial Biology, University of Angers, Angers, France; 2 CNRS UMR 6214, Angers, France; 3 INSERM U1083, Angers, France; 4 CHU d’Angers, Angers, France; The Chinese University of Hong Kong, Hong Kong

## Abstract

Endothelial dysfunction in resistance arteries alters end organ perfusion in type 2 diabetes. Superoxides and cyclooxygenase-2 (COX-2) derivatives have been shown separately to alter endothelium-mediated relaxation in aging and diabetes but their role in the alteration of vascular tone in old diabetic subjects is not clear, especially in resistance arteries. Consequently, we investigated the role of superoxide and COX-2-derivatives on endothelium-dependent relaxation in 3 and 12 month-old Zucker diabetic fatty (ZDF) and lean (LZ) rats. Mesenteric resistance arteries were isolated and vascular tone was investigated using wire-myography. Endothelium (acetylcholine)-dependent relaxation was lower in ZDF than in LZ rats (60 versus 84% maximal relaxation in young rats and 41 versus 69% in old rats). Blocking NO production with L-NAME was less efficient in old than in young rats. L-NAME had no effect in old ZDF rats although eNOS expression level in old ZDF rats was similar to that in old LZ rats. Superoxide level and NADPH-oxidase subunits (p67phox and gp91phox) expression level were greater in ZDF than in LZ rats and were further increased by aging in ZDF rats. In young ZDF rats reducing superoxide level with tempol restored acetylcholine-dependent relaxation to the level of LZ rats. In old ZDF rats tempol improved acetylcholine-dependent relaxation without increasing it to the level of LZ rats. COX-2 (immunolabelling and Western-blot) was present in arteries of ZDF rats and absent in LZ rats. In old ZDF rats arterial COX-2 level was higher than in young ZDF rats. COX-2 blockade with NS398 restored in part acetylcholine-dependent relaxation in arteries of old ZDF rats and the combination of tempol and NS398 fully restored relaxation in control (LZ rats) level. Accordingly, superoxide production and COX-2 derivatives together reduced endothelium-dependent relaxation in old ZDF rats whereas superoxides alone attenuated relaxation in young ZDF or old LZ rats.

## Introduction

Type 2 diabetes is the most frequently encountered metabolic disorder, currently affecting 5% to 10% of most populations, and the incidence continues to grow among developing countries [1]. Associated with obesity, type 2 diabetes is characterized by an insulin resistance inducing metabolic changes such as hyperinsulinemia, hyperglycaemia, dyslipidaemia and hypertension, all leading to increased cardiovascular risk [2]. The morbidity and mortality associated with type 2 diabetes are essentially related to the vascular lesions that develop over time [3]–[5]. Microcirculation is mainly involved, and consequently vital organs such as the brain, heart, kidneys and the limbs are progressively damaged. While the consequences of type 2 diabetes on macrocirculation have been extensively studied [6], [7], much less is known about its effects on microcirculation, especially in old subjects. Aging is associated with a rising incidence of hypertension, obesity and type 2 diabetes; unfortunately the 3 risk factors are usually associated [8]–[10]. Therefore the aim of the present study was to investigate the effect of type 2 diabetes on the endothelium in old rats. Certainly, type 2 diabetes is usually detected late after developing, usually during a medical checkup or when adverse effects induce alerting disorders such as retinal or kidney disorders. Consequently, investigating endothelium-mediated relaxation in older diabetic rats is a key issue.

Type 2 diabetes is associated with an increased reactive oxygen species (ROS) production [11]–[13], which may reduce endothelium-dependent dilation [14], [15]. Similarly, aging is associated with a change in endothelium-dependent dilation with a reduction in NO-dependent dilation in both large and small arteries [16]. The effect of aging is less obvious in small resistance arteries [17], [18]. In resistance arteries ROS may also play a role in the reduction in NO-dependent dilation observed in aging [16]. Furthermore, alteration of the endothelium may also involve an inflammatory process [19]. Consequently, type 2 diabetes associated with aging is likely to induce greater damage to the endothelium. Consequently, we investigated the effect of type 2 diabetes on endothelium-dependent relaxation in young and old rats.

Using Zucker Diabetic Fatty (ZDF) rats, we found that ROS and COX-2 vasoconstrictor derivatives additionally reduced endothelium-dependent relaxation in old diabetic rats whereas ROS alone are involved in the decrease in endothelium-dependent relaxation in young ZDF or in old LZ rats.

## Methods

### Animals

Adult male Zucker fatty diabetic (ZDF) and lean Zucker (LZ) rats, 12–14 and 50–52 week-old, were purchased from Charles River (L’Arbresles, France). Rats were anesthetized (Isoflurane, 2.5%) and the right femoral artery was catheterized for blood pressure measurement [20]. Animals were then sacrificed by CO2 inhalation, the gut excised and mesenteric arteries gently dissected. From each rat, several arterial segments (second order mesenteric arteries similar in size) were isolated and used for pharmacology, biochemical and immuno-histological analyses. Before sacrifice, a sample of blood was collected for analyses [21].

In a separated series of experiments 12-month-old male LZ and ZDF rats were treated with the COX2 inhibitor Celecoxib (25 mg/kg/day, forced feeding once a day, 21 days) or with the combination of Celecoxib (25 mg/kg/day) plus 1-Oxyl-2, 2, 6, 6-tetramethyl-4-hydroxypiperidine (tempol, forced feeding once a day, 10 mg/kg per day, 21 days) [12], [22].

The procedure followed in the care and euthanasia of the study animals complied with the European Community Standards on the Care and Use of Laboratory Animals (Ministère de l’Agriculture, France, authorization No. 6422). The protocol was approved by the Committee on the Ethics of Animal Experiments of the “Pays de la Loire” Region (“Comité d’éthique en Expérimentation Animale”, CEEA, permit # CEEA PdL 2008.10).

### Pharmacological Profile of Isolated Mesenteric Arteries

Segments of mesenteric arteries (2 mm long) were mounted on a wire-myograph (DMT, Aarhus, DK) [23]. 2 tungsten wires (40 µm diameter) were inserted into the lumen of the arteries and respectively fixed to a force transducer and a micrometer. Arteries were bathed in a physiological salt solution (PSS) of the following composition (mmol/L): 130.0, NaCl; 15.0, NaHCO3; 3.7, KCl; 1.6, CaCl2; 1.2, MgSO4 and 11.0, glucose (pH 7.4, PO2 160 mm Hg, and PCO2 37 mm Hg) [24]. Wall tension was applied as described previously [25]. Artery viability was tested using a potassium rich PSS (80 mmol/L). Cumulative concentration-response curve to phenylephrine (1 nmol/L to 10 µmol/L) was then performed [26]. After washout the arteries were precontracted with phenylephrine to a level approximately equivalent to 50% of the maximal response. Cumulative concentration-response curve to acetylcholine (ACh, 0.001 to 3 µmol/L) was then performed in the presence or absence of N (omega)-nitro-L-arginin methyl ester (L-NAME, 100 µmol/L, 20 min), tempol (10 µmol/L, 20 min) or N- [2- (cyclohexyloxy)-4-nitrophenyl]-methanesulfonamide (NS398, 10 µmol/L, 20 min) for 20 min [27].

### Western Blot Analysis

Other segments of arteries were homogenized and proteins (25 µg total protein from each sample) were separated by SDS-PAGE using a 4% stacking gel followed by a 10% running gel. Proteins were detected with specific antibodies (Transduction Laboratories, eNOS 1∶1000, Cav-1 1∶4000, p67 and gp91 and actin 1∶1000 in T-TBS-BSA 5%). COX-2 polyclonal antibodies (1∶1000) were obtained from Santa Cruz Biotechnology. Protein expression was visualized using the ECL-Plus Chemiluminescence kit (Amersham) [28].

### Detection of Reactive Oxygen Species (ROS) Using Confocal Microscopy

Other arterial segments were embedded vertically in Tissue-tek (Sakura) and frozen in isopentane. ROS detection was performed on transverse cross sections 7 µm thick incubated with dihydroethydine (DHE) as previously described [29]. DHE, in the presence of superoxide, is briefly oxidized to fluorescent ethidium bromide. Ethidium bromide is trapped by intercalation with DNA, and the number of fluorescent nuclei indicates the relative level of superoxide production. In negative control experiments arterial sections were incubated with Tempol before DHE staining [29]. Positive control experiments were performed using MRA isolated from a rat treated for 6 hours with bacterial lipopolysacharide. Positive staining was visualized using confocal microscopy and QED-image software (Solamere Technology, Salt Lake City, UT) [30]. Image analysis was performed using Histolab (Microvision, France) [31].

### Immunohistological Analysis of COX-2

Immunostaining of COX-2 was performed as previously described [12] segments of mesenteric resistance arteries were mounted in embedding medium (Tissu-Tek, Miles, Inc), frozen in isopentane, pre-cooled in liquid nitrogen and stored at −80°C. Transverse cross sections (7 µm thick) were incubated with primary goat anti-COX-2 polyclonal antibodies (Santa Cruz Biotechnology, 1∶100) followed by the fluorescent secondary antibody (1∶200). In negative control experiments the primary antibody was omitted. Positive control experiments were performed using MRA isolated from a rat treated for 6 hours with bacterial lipopolysacharide. Positive staining was visualized as described above [32].

### Statistical Analysis

Results are expressed as means ± SEM. Significance of the difference between arteries was determined by ANOVA (1-factor ANOVA or ANOVA for consecutive measurements, when appropriate). Means were compared by paired t-test or by the Bonferroni test for multigroup comparisons. Values of p<0.05 were considered to be significant.

## Results

### Animals Parameters

Rat body weight, greater in young ZDF rats than in young LZ rats decreased with age in ZDF rats (table 1), in agreement with previous studies [33], [34]. Nevertheless, blood glucose in ZDF rats was significantly higher than in LZ rats and was greater in old ZDF rats. ZDF rats were slightly, but significantly hypertensive (table 1).

**Table 1:** Rat body weight, glycaemia and mean arterial blood pressure (MAP) were measured in 3- and 12-month old LZ and ZDF rats.

	3-month old rats		12-month old rats	
	LZ rats	ZDF rats	LZ rats	ZDF rats
Body weight (g)	325±11	441±17*	567±28#	382±10*#
Glycaemia (mg/dL)	112±13	302±23*	125±11	421±37*#
MAP (mmHg)	98±4	108±6	112±5	132±6*#

Mean±SEM is presented (n = 12 rats per group).

* P<0.05, ZDF versus LZ of the same age.

# P<0.05, effect of age within each group.

### Endothelium-dependent Relaxation of Mesenteric Resistance Arteries

**Figure 1:**
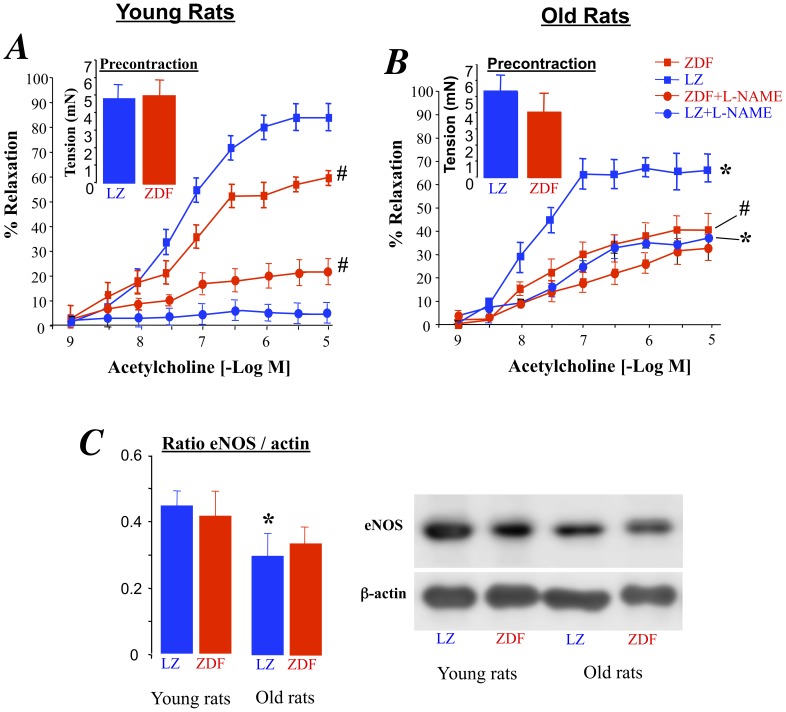
Acetylcholine-induced relaxation in young (A) and old (B) LZ and ZDF rats. Relaxation was obtained before and after NO synthesis inhibition with the NO synthesis blocker L-NAME. The inserts show the precontraction applied before adding acetylcholine. Beta-actin and eNOS expression levels were measured using Western-Blot analysis (typical blots on the right side). Data is given as a ratio eNOS/beta-actin (**C**). Mean ± SEM is presented (n = 10 per group). #P<0.01, ZDF versus LZ. *P<0.01, old versus young rats.

Acetylcholine-dependent relaxation was reduced in ZDF rats compared to lean rats (figure 1A–B). Furthermore, aging per se was also associated with a significant reduction in acetylcholine-mediated relaxation in both LZ and ZDF rats (figure 1A–B). The reduction in relaxation due to ageing was greater in ZDF rats than in LZ rats (47±6% in old rats versus 28±4% in young rats, P<0.01), showing that the combination of diabetes and aging further impaired endothelium-dependent relaxation. Precontraction prior to acetylcholine-mediated relaxation was similar in the different groups (inserts in figure 1 A and B).

In young LZ rats, L-NAME suppressed acetylcholine-mediated relaxation whereas it was inhibited, but not suppressed, in old LZ rats (figure 1B). In ZDF rats, L-NAME significantly reduced acetylcholine-induced relaxation by 61±8% in young rats (figure 1A) and by only 24±4% in old rats (figure 1B, P<0.01 versus young ZDF rats). Accordingly the inhibitory effect of L-NAME was greatly reduced in old ZDF rats. Furthermore, eNOS expression level (relative to beta-actin) was lower in older rats than in young rats, although no difference between ZDF and LZ rats was found in the mesenteric resistance arteries (figure 1C). Finally, eNOS expression level, reduced in old versus young rats, was not altered by type 2 diabetes.

### Reactive Oxygen Species Detection and Effect on Endothelium-dependent Relaxation

**Figure 2:**
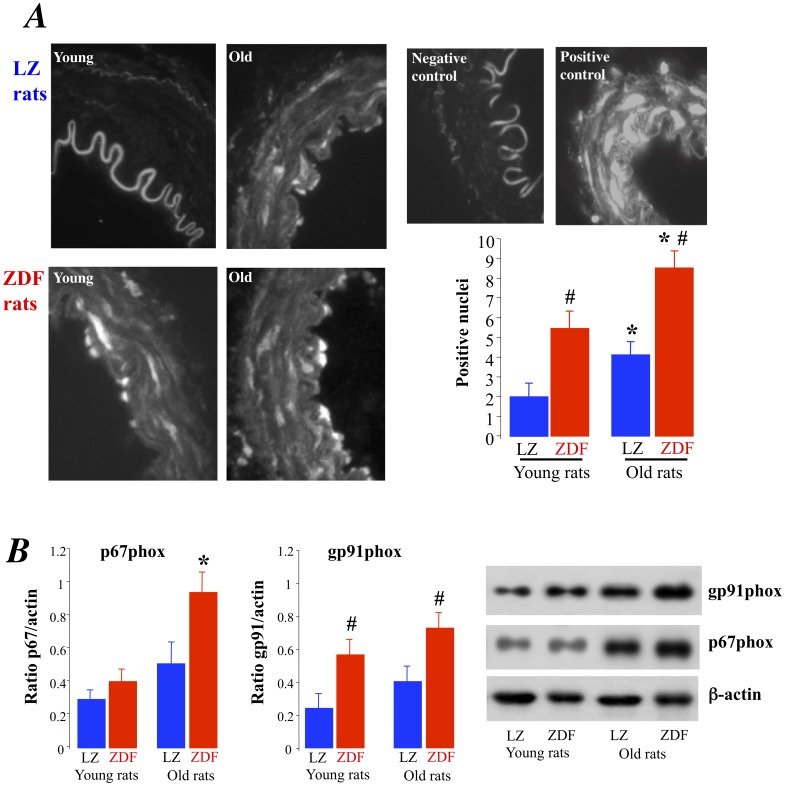
Measurement of the reactive oxygen species level in mesenteric resistance arteries isolated from LZ and ZDF rats using dihydroethydin microfluorography (A). The expression level of the NADP(H)-oxidase subunits gp91 and p67 (B) was measured using Western-Blot analysis. Mean ± SEM is presented (n = 10 per group). #P<0.01, ZDF versus LZ. *P<0.01, old versus young rats.

**Figure 3:**
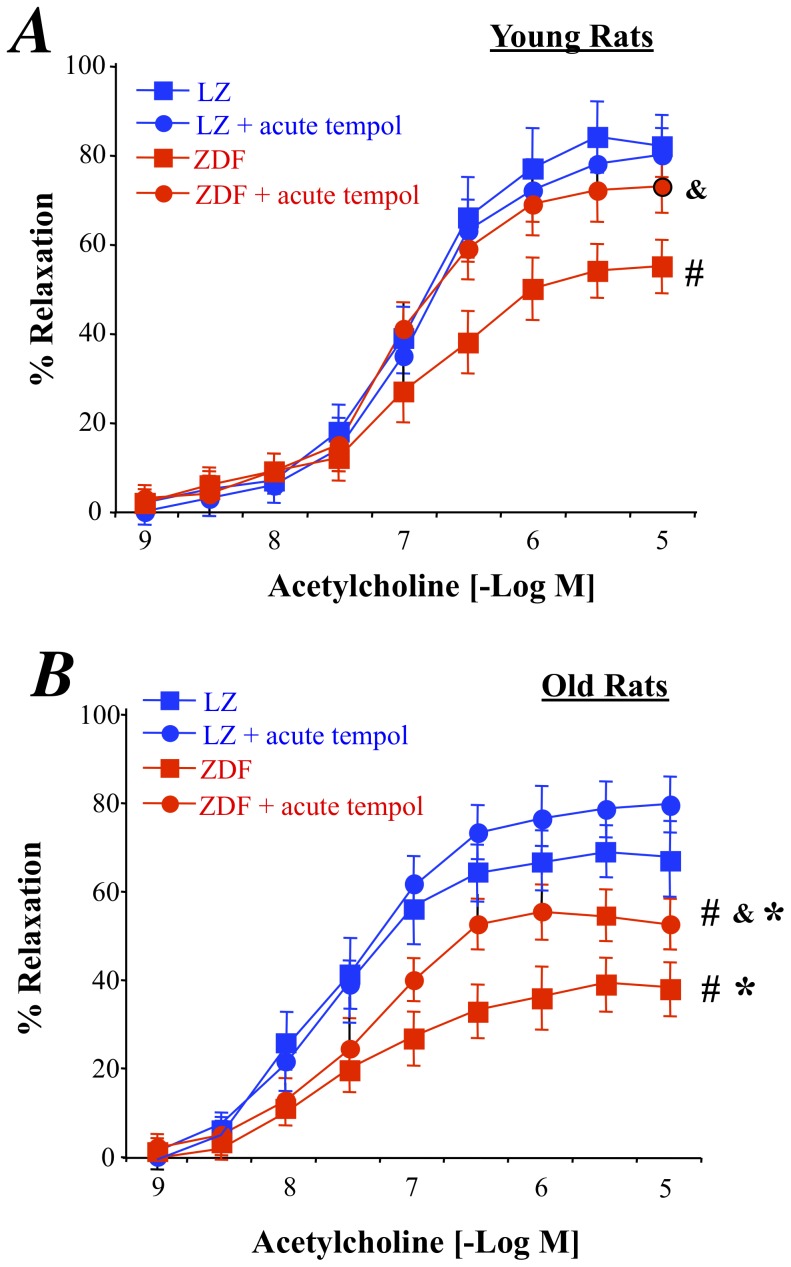
Effect of the acute treatment of mesenteric resistance arteries with the antioxydant tempol on acetylcholine-induced relaxation in mesenteric resistance arteries isolated from young (A) and old (B) LZ and ZDF rats. Mean ± SEM is presented (n = 10 per group). #P<0.01, ZDF versus LZ. *P<0.01, old versus young rats. &P<0.05, effect of tempol on acetylcholine-induced relaxation.

As excessive oxidative stress has been shown to reduce endothelium-mediated dilation in diabetes, we investigated the ROS level in mesenteric arteries was analyzed using dihydroethidin (DHE) microfluorography. We found that DHE staining was higher in ZDF than in LZ rats whatever the age. In addition, in older animals the ROS level was higher than in younger animals in both LZ and ZDF rats (figure 2A). The increased DHE staining was associated with a greater expression level of the NAD(P)H-oxidase subunit p67phox in old ZDF rats than in the other groups (figure 2B) whereas the gp91phox expression level was significantly higher in both young and old ZDF rats compared to age-matched LZ rats (figure 2B). Furthermore, tempol, acutely added to the bath of isolated mesenteric arteries improved acetylcholine-induced relaxation in both young and old ZDF rats without affecting relaxation in LZ rats (figure 3). In young ZDF rats, acetylcholine-induced relaxation in the presence of tempol was equivalent to the relaxation found in LZ rats (figure 3A). On the other hand, although tempol significantly improved relaxation in old ZDF rats, it did not fully restore relaxation to the level found in LZ rats (figure 3B) suggesting that another vasoconstrictor agent prevented complete relaxation.

### COX-2 Detection and Effect on Endothelium-dependent Relaxation

**Figure 4:**
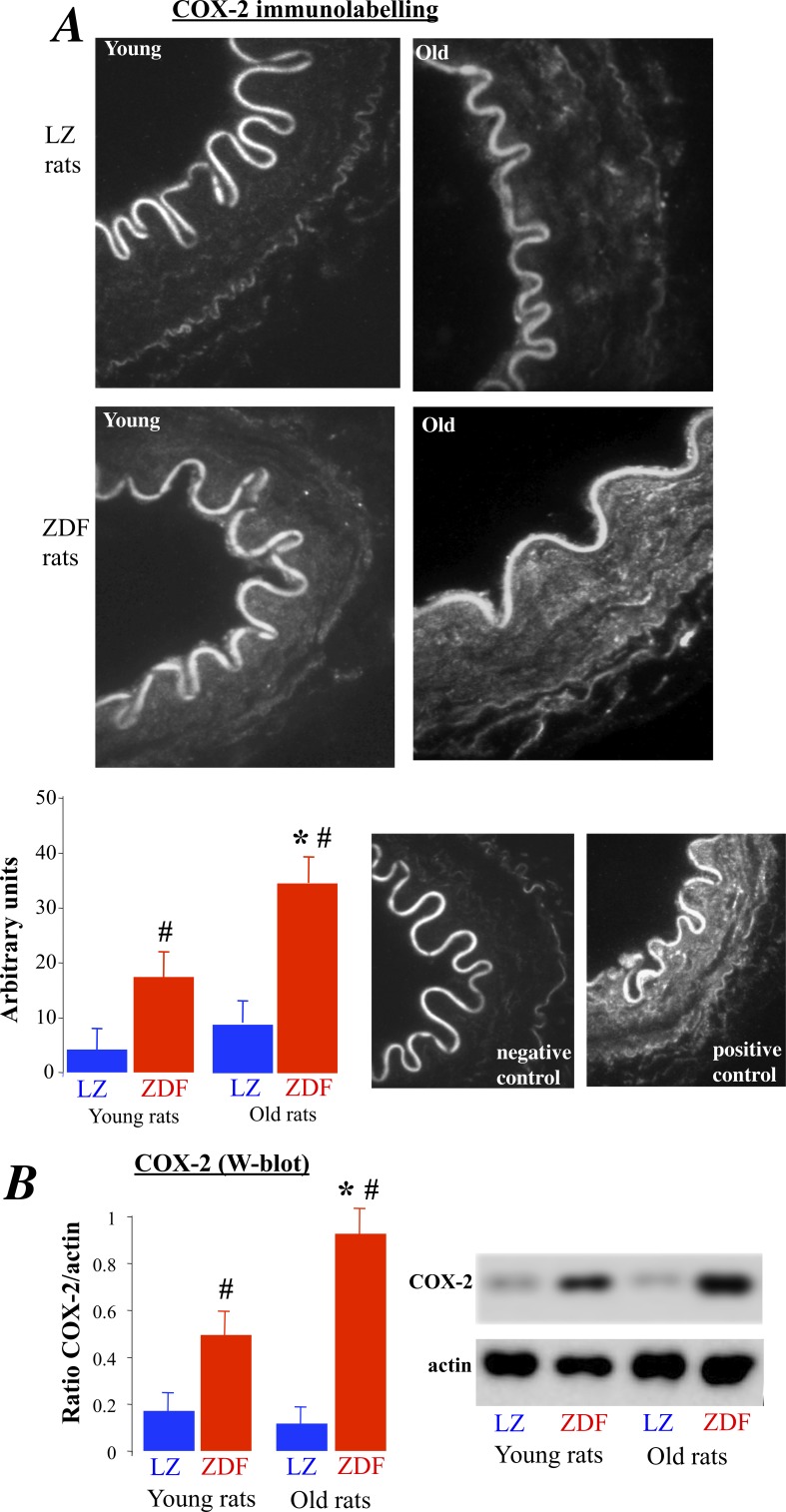
Measurement of COX-2 expression level in mesenteric resistance arteries isolated from young and old LZ and ZDF rats using immunolabelling (A) and Western-blot analysis (B). Mean ± SEM is presented (n = 10 per group). #P<0.01, ZDF versus LZ. *P<0.01, old versus young rats.

**Figure 5:**
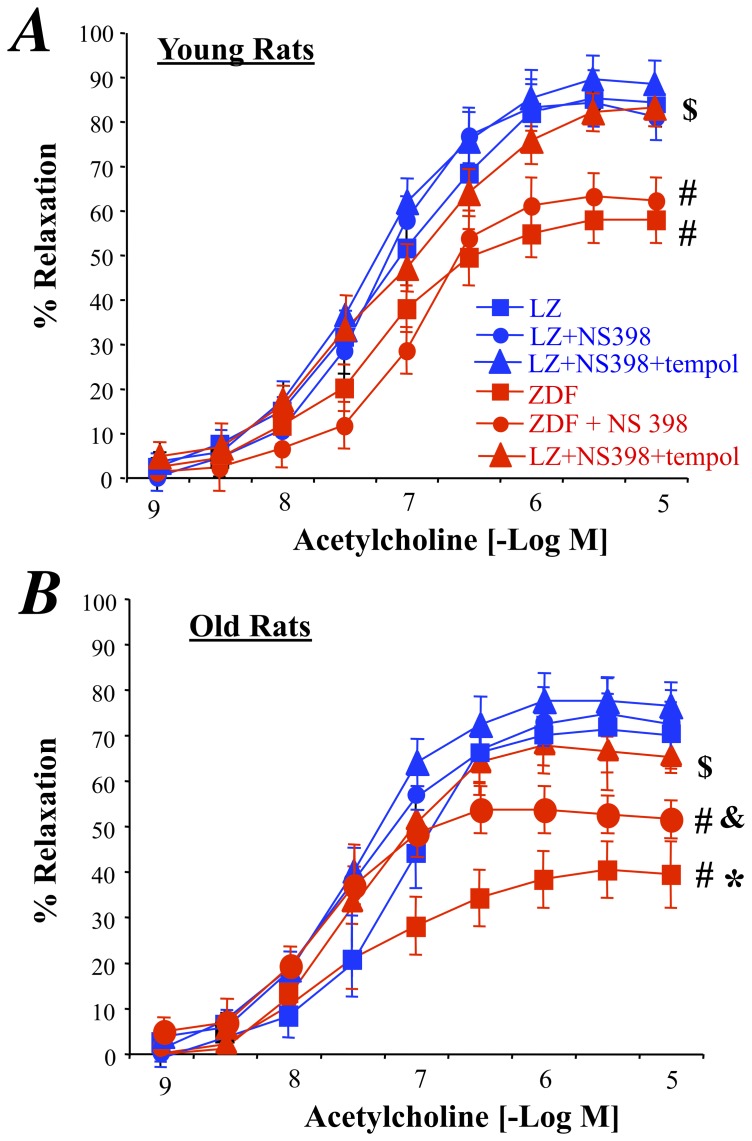
Effect of an acute treatment of mesenteric resistance arteries with the COX-2 inhibitor NS398 or NS398 and the antioxidant tempol on acetylcholine-induced relaxation in young (A) and old (B) LZ and ZDF rats. Mean ± SEM is presented (n = 10 per group). ^#^P<0.01, ZDF versus LZ. *P<0.01, old versus young rats. ^&^P<0.05, effect of NS398 alone on acetylcholine-induced relaxation. ^$^P<0.05, effect of NS398+tempol on acetylcholine-induced relaxation.

COX-2 was expressed in ZDF rats and not in LZ animals (figure 4A and B). In addition, in old rats COX-2 expression level was significantly greater than in young ZDF rats. In order to determine the role of COX-2 in the endothelium dysfunction, we tested the effect of the COX-2 inhibitor NS398 on acetylcholine-induced relaxation in the arteries of ZDF rats (figure 5). First, NS398 had no significant effect on acetylcholine-induced relaxation in young and old LZ rats. Similarly, NS398 did not affect acetylcholine-induced relaxation in young ZDF rats (figure 5A). In young ZDF rats, NS398 and tempol improved relaxation so that it was no different than LZ rats. By contrast with young ZDF rats, NS398 in old ZDF rats significantly improved acetylcholine-induced relaxation (figure 5B), although relaxation in the presence of NS 398 remained significantly lower than in LZ rats. The addition of tempol to NS398 fully improved acetylcholine-induced relaxation in old ZDF rats, so that acetylcholine-induced relaxation in the presence of NS398 and tempol was no different than in LZ rats. Accordingly, in old ZDF COX-2 derived vasoconstrictor prostanoids was associated with superoxide in order to further reduce endothelium-dependent relaxation.

### Phenylephrine-mediated Contraction and Endothelium-independent Relaxation

**Figure 6:**
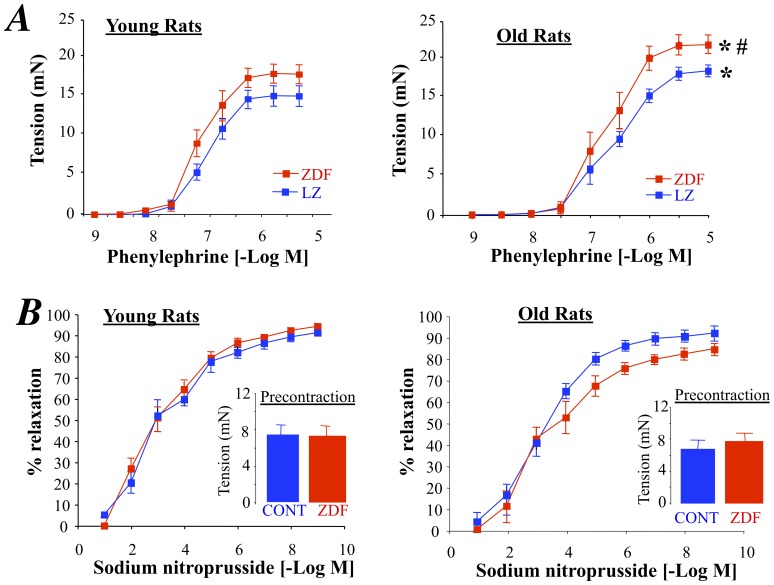
Concentration-response curves to phenylephrine (A) and sodium nitroprusside (B) performed in isolated arteries from young and old LZ and ZDF rats. The inserts in panels B represent the precontraction applied to arteries before sodium nitroprusside-mediated relaxation. Mean ± SEM is presented (n = 10 per group). *P<0.05, old versus young rats ^#^P<0.05, ZDF versus LZ rats.

Finally, in old rats phenylephrine-mediated contraction was greater than in young rats (figure 6A). The contraction was also higher in ZDF than in LZ rats, although the difference only reached significance in old rats (figure 6A). On the other hand, sodium nitroprusside, which induced endothelium-independent relaxation, was not affected by aging or by diabetes (figure 6B).

### In vivo Blockade of COX-2

**Figure 7:**
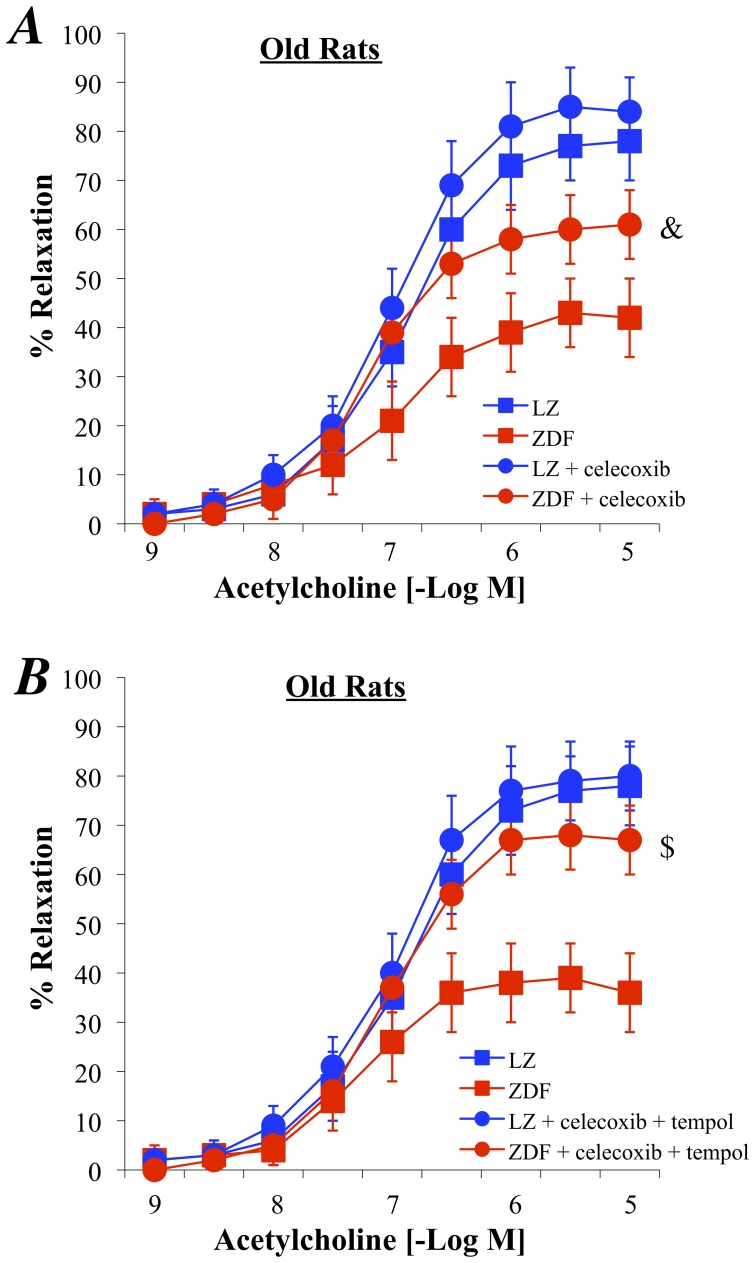
Effect of a chronic treatment of old LZ and ZDF rats with celecoxib (A) or with the combination of celecoxib and tempol (B) on acetylcholine-induced relaxation of isolated mesenteric resistance arteries. Mean ± SEM is presented (n = 8 per group). ^&^P<0.05, effect of celecoxib alone on acetylcholine-induced relaxation. ^$^P<0.05, effect of celecoxib+tempol on acetylcholine-induced relaxation.

In old ZDF rats treated chronically with the COX-2 inhibitor celecoxib, acetylcholine-induced relaxation was improved whereas celecoxib did not affect relaxation in old LZ rats (figure 7A). Nevertheless, acetylcholine-induced relaxation in old ZDF rats treated with celecoxib did not reach the level of relaxation obtained in LZ rats (figure 7A). In old ZDF rats treated chronically with the celecoxib plus the antioxidant tempol, acetylcholine-induced relaxation was improved and reached the level obtained in LZ rats (figure 7B). The treatment with celecoxib plus tempol did not affect relaxation in old LZ rats (figure 7B).

## Discussion

In this study we found that endothelium-dependent relaxation was reduced in young rats suffering from type 2 diabetes due to excessive ROS production, whereas in one year-old diabetic rats COX-2 derivatives in association with an excessive ROS production reduced endothelium dependent relaxation.

First, we found that endothelium-mediated dilation, evidenced by acetylcholine-dependent relaxation was reduced in ZDF rats compared to lean rats. This finding is in agreement with previous studies showing that type 2 diabetes is associated with endothelial dysfunction in rats [35] as well as in humans [36]. Furthermeore, aging per se was associated with a significant reduction in acetylcholine-mediated relaxation in both LZ and ZDF rats, in agreement with previous studies in large conduit arteries [37] and in small resistance arteries [12], [35], [38]. Also in agreement with these previous works, we found that the combination of diabetes and aging further impaired endothelium-dependent relaxation.

As endothelium-mediated relaxation in mesenteric arteries relies greatly on the production of NO [39]–[42], concentration-response curves to acetylcholine were repeated after incubation arterial with L-NAME. Although L-NAME significantly reduced relaxation in all groups, the inhibitory effect of L-NAME was greatly reduced in old ZDF rats suggesting a reduced involvement of NO in the relaxation. Thus, in order to find the origin of the excessive endothelial dysfunction found in old ZDF rats we measured eNOS expression level in the mesenteric artery in the 4 study groups. Nevertheless, we found no difference between ZDF and LZ rats. This observation is in agreement with previous studies of resistance arteries [38], [43]–[45] and of large arteries [46] showing a reduced NO-dependent relaxation in diabetic rats. Similarly, NO-mediated dilation is also reduced in humans [11], [47], [48]. Surprisingly, eNOS expression level, reduced in old versus young rats, was not altered by type 2 diabetes. This is in agreement with previous studies performed on mesenteric arteries isolated from diabetic db/db mice, another model of type 2 diabetes [49]. On the other hand, in streptozotocine-induced diabetes, eNOS expression decreases together with a reduced NO production [50]. In human subcutaneous arterioles, obesity associated with diabetes or not, was accompanied by a reduction in eNOS expression level and endothelium-mediated dilation [51]. Indeed, it is most likely that NO bioavailability is reduced in diabetes mainly due to excessive oxidative stress. Dimerization of the enzyme is a key step in the production of NO [13], [52] and eNOS dimerization is strongly reduced in ZDF rats’ mesenteric arteries [53]. The authors have shown that NO-mediated dilation was reduced due to increased oxidative stress leading to eNOS uncoupling rather than to a reduction in expression level.

As excessive oxidative stress has a major role in the endothelium dysfunction observed in diabetes, we investigated the role of superoxide in acetylcholine-mediated relaxation. Certainly, oxidative stress has been shown to play a major role in the reduction in endothelium-mediated relaxation in diabetes in most vascular territories [54] including the mesenteric vasculature [12], [49]. A similar superoxide effect has been shown in the metabolic syndrome [10], [21], [22], [55], [56]. In the present study the ROS level in mesenteric arteries was analyzed using dihydroethidin (DHE) microfluorography as previously shown [21], [22], [57]. We have previously validated this approach in the detection of ROS in isolated mesenteric arteries. Certainly, we obtained similar results with DHE staining as with rhodamin-staining, 3-nitrotyrosine labeling, NADP(H)Oxidase subunits level measurement and O2- quantification using electron paramagnetic resonance [29]. In the present study, DHE staining was larger in ZDF than in LZ rats whatever the age and it was higher in old than in young animals in both LZ and ZDF rats. The increased DHE staining was associated with a greater expression level of the NAD(P)H-oxidase subunits in old ZDF rats. The occurrence of an excessive oxidative stress reducing endothelium (NO)-dependent relaxation was confirmed using the acute effect of the antioxidant tempol as previously shown and validated [21], [29], [54]. This test is indeed a functional proof of the effect of ROS on endothelium-mediated relaxation. Acute tempol improved acetylcholine-induced relaxation in both young and old ZDF rats without affecting relaxation in LZ rats whereas in young ZDF rats, acetylcholine-induced relaxation in the presence of tempol was equivalent to the relaxation found in LZ rats. This observation shows that ROS reduced endothelium (NO)-mediated relaxation in both young and old ZDF rats. This is in agreement with previous studies showing that ROS reduces endothelium-mediated relaxation in ZDF rats as well as in other animal models of type 2 diabetes [15], [45], [53], [58] and in human endothelial cells [59]. Nevertheless, in old ZDF rats, tempol did not fully restore relaxation to the level found in LZ rats suggesting that another vasoconstrictor agent was involved.

In order to better understand this endothelial dysfunction found in old ZDF rats, we investigated the involvement of COX-2 in acetylcholine-induced relaxation. Indeed, COX-2 is expressed in resistance arteries [22], [60], [61] as well as in large vessels [62] in animal models of ageing. In these studies, COX-2 expression was associated with the production of vasoconstrictor prostanoids. In the present study, COX-2 was detected using immunostaining and Western-Blot in ZDF rats and not in LZ animals. Furthermore, in old ZDF rats COX-2 expression was significantly larger than in young ZDF rats. In agreement with this observation, we found that NS398 had no effect on acetylcholine-induced relaxation in young and old LZ rats as well as in young ZDF rats. By contrast, NS398 in old ZDF rats significantly improved acetylcholine-induced relaxation, although relaxation in the presence of NS 398 remained significantly lower than in LZ rats. The addition of tempol to NS398 fully improved acetylcholine-induced relaxation in old ZDF rats, so that acetylcholine-induced relaxation in the presence of NS398 and tempol was no different than in LZ rats. Accordingly, in old ZDF COX-2 derived vasoconstrictor prostanoids was associated with superoxide in order to further reduce endothelium-dependent relaxation. As COX-2 derived vasoconstrictors were involved in old ZDF rats which also have a greater oxidative stress a possible explanation could be that ROS per se activate COX-2, at least in part. Indeed, a recent study has clearly shown that oxidative stress has a key role in the increased COX-2 activity observed in renal arteries of renovascular hypertensive rats. Indeed, this later study also shows that COX-2-derived PGF_2α_ has an major role in mediating endothelial dysfunction [63]. The involvement of ROS and COX-2 derivatives was further confirmed using old ZDF rats treated chronically with the COX-2 inhibitor celecoxib alone or in combination with tempol. Endothelium-mediated relaxation was fully restored to control level only when the 2 substances were given together to the rats. Nevertheless, it should be noted that the use of COX-2 inhibitors is not recommended as these drugs possess deleterious side effects including higher risk of myocardial infaction [64].

Finally, in old rats phenylephrine-mediated contraction was greater than in young rats. Furthermore, phenylephrine-mediated contraction was also higher in ZDF than in LZ rats. These observations are in agreement with previous studies, which have shown that both aging [37], [65] and diabetes [12], [66] are associated with arterial wall hypertrophy and consequently with hypercontractility.

## Conclusion

We found that long-term exposure to diabetes increased endothelium damages and further reduced endothelium-mediated relaxation in mesenteric resistance arteries. In these arteries COX-2 derivatives in addition to ROS contributed to severely reduce endothelium-dependent dilation. This finding is of importance as it shows that deleterious pathways may add one to each other when diabetes evolves over time. Certainly, the present experimental conditions most probably represent a more common pathological condition as diabetes is usually diagnosed several months or years after the onset of the metabolic disorders leading to hyperglycemia. Consequently, the present study reinforces the assumption that not only oxidative stress but also inflammation contribute, most probably synergistically, to the vascular disorders occurring in type 2 diabetes.
